# Should I Take Prediabetes Seriously or Not: A Qualitative Study on People's Perceptions of Prediabetes

**DOI:** 10.1155/jdr/8063481

**Published:** 2025-02-12

**Authors:** Katri Harcke, Marit Graue, Timothy Charles Skinner, Christina B. Olsson, Dan Grabowski, Nouha Saleh-Stattin

**Affiliations:** ^1^Department of Neurobiology, Care Sciences and Society, Division of Family Medicine and Primary Care, Karolinska Institutet, Huddinge, Sweden; ^2^Academic Primary Health Care Centre, Region Stockholm, Stockholm, Sweden; ^3^Department of Health and Caring Sciences, Western Norway University of Applied Sciences, Bergen, Norway; ^4^Institute of Psychology, University of Copenhagen, Copenhagen, Denmark; ^5^Australian Centre for Behavioural Research in Diabetes, Institute for Health Transformation, Melbourne, Australia; ^6^Department of Neurobiology, Care Sciences and Society, Division of Physiotherapy, Karolinska Institutet, Huddinge, Sweden; ^7^Department of Prevention, Health Promotion and Community Care, Copenhagen University Hospital-Steno Diabetes Center Copenhagen, Herlev, Denmark

**Keywords:** person-centered care, prediabetes, primary healthcare, primary prevention

## Abstract

It is critical to ensure that lifestyle change programs are tailored to the person with prediabetes needs and wishes. However, programs that are carried out in research settings to delay or prevent Type 2 diabetes in people with prediabetes do not translate easily to everyday settings. There is a need to explore further the perceptions of people with prediabetes about the condition and their role in self-management to better balance the content of intervention programs for prediabetes with the participants' life context and experience. For this purpose, we invited 21 persons with prediabetes from four primary healthcare centers in Region Stockholm, Sweden, for individual interviews. Transcripts were analyzed with qualitative content analysis. Two main themes were identified, *prediabetes: a condition between health and disease* and *I must manage prediabetes myself but need support*. This in-between state has a serious impact on the decisions that people with prediabetes make concerning self-management and behavioral changes. One of the main findings of this study highlights the importance of communicating the diagnosis of prediabetes clearly and the importance of preventive actions as this can trigger behavioral change. People with prediabetes in our study shed light on different needs for support to make and maintain behavioral change which requires a person-centered approach. This support was described internally, from family and peers, or externally from healthcare professionals. These results will be used in a codesign study where healthcare professionals and persons with prediabetes discuss the components of a person-centered model for a behavioral change intervention in primary healthcare.

## 1. Introduction

Prediabetes is a high-risk state for the development of Type 2 diabetes. It is defined as having elevated blood glucose levels, impaired glucose tolerance (IGT) and/or impaired fasting glucose (IFG) but below the diagnostic threshold for Type 2 diabetes [1, 2]. Research has shown that about 70% of individuals with prediabetes will develop Type 2 diabetes in their lifetime if actions to prevent it are not taken [3, 4]. Due to differences in the criteria used to define “prediabetes” around the world, it is hard to estimate how many have the condition. However, according to the International Diabetes Federation (IDF) in 2021, 541 million adults were estimated to have IGT. By 2045, this figure is projected to increase to 730 million adults in the world. The number for IFG was 319 million adults and is estimated to rise to 441 million adults in 2045 [4].

In the early 2000s, several intervention studies tested the effectiveness of lifestyle interventions to prevent individuals with prediabetes progressing to diabetes [5–7]. These studies showed that lifestyle interventions focusing on healthier eating habits and moderate exercise could reduce the risk of Type 2 diabetes by 58%. Based on these research results, international organizations have recommended behavioral change as the main treatment for prediabetes [1, 2]. Some countries, such as the United States and Great Britain have developed and are implementing diabetes prevention programs that support health behavior change. Regardless, research shows that most people with prediabetes are not being referred to these prevention programs or have dropped out after initial attendance [8, 9]. In a review study by Aziz et al. [10], they argued that the low participation in these diabetes prevention programs could be that the focus was on the content of the interventions, instead of balancing the content with the participants' life context and experience, making the participants disengaged from the intervention.

A recent qualitative metasynthesis study [11] on self-management and perceptions of prediabetes highlighted that it is critical to ensure that interventions are suited to the person's needs. Further, Howell et al.'s [12] study, exploring the perception of risk in people with prediabetes, concluded that persons were hovering between a state of health and disease, which could affect their engagement and participation in behavioral change and prevention programs. Therefore, before codesigning an intervention in primary healthcare centers for people with prediabetes together with healthcare professionals, there is a need to further explore the perceptions of people with prediabetes about the condition and their involvement in their self-management of prediabetes.

## 2. Aims

This study is aimed at exploring and describing how people with prediabetes perceive having prediabetes and the support they need to manage and sustain behavioral changes that could decrease the risk of developing Type 2 diabetes.

## 3. Method

### 3.1. Design and Setting

In this qualitative study, data were collected in Region Stockholm between 2022 and 2023 in four primary healthcare centers. The interviews were either digital or face to face. Digital interviews were carried out by phone, through Zoom or Teams. The face-to-face interviews took place either at the residence of the interviewees or at the researcher's (K.H.) working place, the Academic Primary Health Care Centre, according to the participants' wishes.

### 3.2. Participants

Diabetes nurses working at primary healthcare centers in Region Stockholm and their managers were asked, to invite persons with prediabetes to participate in this interview study. The inclusion criteria were adults (18 and over) who had prediabetes diagnosis. Exclusion criteria were those who already had developed Type 2 diabetes. Information about the study and consent forms were supplied to persons with prediabetes for participating. Persons with prediabetes who agreed to be part of the study signed the consent form and were then contacted by the researcher to schedule the interviews. Twenty-one persons from four different primary healthcare centers across Region Stockholm were interviewed. The interviewer/researcher had no previous contact with the participants.

### 3.3. Data Collection

The researchers developed a semistructured interview guide, based on the study aims and research questions (Supporting Information 1 Interview guide). Six interviews were carried out digitally and, 15 were face to face. The interviews ranged from 8 to 45 min, were audio recorded, and were transcribed verbatim. All interviews were conducted individually in Swedish, by K.H. who had previous experience conducting similar interviews in other studies. The first individuals who agreed to participate had similar characteristics: the majority were 65 years old and above and were mostly retired. Thus, we opted to invite six younger persons with prediabetes, who were still working from the same four primary healthcare centers.

### 3.4. Data Analysis

Interview transcripts were analyzed with qualitative content analysis [13] in Swedish. The first author (K.H.) conducted the initial analysis, thoroughly and repeatedly rereading the transcripts to achieve an in-depth understanding. This was followed by extracting the relevant parts to gain a clearer picture and identifying meaning units. The meaning units were further condensed and labeled with codes. This was initially done separately by K.H. and N.S.-S. and then together in dialogue. The codes were then grouped into subthemes and further two main themes emerged. All the coauthors discussed the findings. Quotes from the transcripts were used to demonstrate credibility and authenticity to the results as described by Lindgren, Graneheim, and Lundman [13]. The quotes were translated to English by K.H. and checked by N.S.-S. The SRQR checklist [14] guided the reporting.

## 4. Results

### 4.1. Participants' Characteristics

Twenty-one persons with prediabetes agreed to participate in the study. There were 11 women and 10 men, aged between 28 and 85, with a median age of 69 years. All had prediabetes diagnoses ranging from 1 to 20 years (Table 1).

### 4.2. Main Themes and Subthemes

The analysis of the interviews revealed two main themes (Table 2), prediabetes: a condition between health and disease and I must manage prediabetes myself but need support.

#### 4.2.1. Prediabetes: A Condition Between Health and Disease

The first main theme, prediabetes: a condition between health and disease, came from how people with prediabetes described the situation when they were told that they had prediabetes and their reflections on the diagnosis. This theme was inspired by the subthemes: *the in-between perception of prediabetes* and *prediabetes is my opportunity not to develop Type 2 diabetes*.

##### 4.2.1.1. The In-Between Perception of Prediabetes

Upon initially receiving the prediabetes diagnosis, different responses were recounted by the participants. An important factor was how the healthcare professionals delivered the diagnosis, which impacted persons with prediabetes's understanding of the risk and severity of the condition. For some, when healthcare professionals used words like *“close to the limit”* or *“slightly high sugar values,”* this led even to uncertainty about having prediabetes or not.

…so I think that when they told me that... that “yes, you have little high sugar levels”, they did not convey that there was any greater risk. “Yeah, yeah.” They could have said instead “Well, if you don't take care of yourself, you can get diabetes” because then I would have taken it much more seriously, but I didn't really take it in. (Person 2)

No one has told me that I really have prediabetes. … The nurse said, close to the limit. (Person 17)

[When I was diagnosed, the doctor said to me] “if you want to avoid medication and other things, all you have to do is ... that you exercise and change your diet”. And that's kind of what I needed to hear. (Person 5)

Having prediabetes was described by some participants as of no concern, especially when compared to other illnesses or important life events. Others did not even perceive it as a disease at all.

…I don't think about diabetes at all. There are other things to think about before. … It doesn't make my life a problem. (Person 11)

On the other hand, some associated it with Type 2 diabetes, its complications, and heredity and wanted to make changes to healthier habits to prevent it.

...neither I nor my mother knew that this could be, like a hereditary gene so to speak. However, we knew that my aunt had developed diabetes and an uncle of theirs. Then [when I got the diagnosis] I felt like this, well, if I still feel good, I'll let it be. But then it felt like it hung over my head and I felt that I need to take control over this ... And then I also thinking like, I have a son... And it's also for his sake... On one hand, you want to keep track of your blood sugar levels for your own sake, but maybe also like considering that if it is...hereditary in the genes, it could be something that he too could be affected in some way. (Person 13)

The prediabetes diagnosis was also related to preconception of the condition and therefore to different reactions when receiving the diagnosis. Some had foreseen it because peers or family members have it; thus, they had previous knowledge about prediabetes and Type 2 diabetes.

Since quite a lot of my peers…have it… and type 2 diabetes… and high blood pressure that needs treatment, so like… Yeah,… So I wasn't that surprised. (Person 5)

Others were caught off guard by the diagnosis as they perceived themselves as already having healthy food choices and pursuing physical activities, even though for some they had Type 2 diabetes in the family.

…I was mostly surprised because I did not feel any symptoms or anything since I think I'm careful with anything that contains sugar and is quite physically active… (Person 21)

##### 4.2.1.2. Prediabetes Is My Opportunity Not to Develop Type 2 Diabetes

For some of the participants, just being told about the prediabetes diagnosis triggered a response. It was a wake-up call, increasing their awareness and giving them a nudge to improve self-management. Some expressed it as “*a need to pull oneself together*,” others “*to take responsibility for self-care*,” and some “*sees no obstacles*” for self-management. The prediabetes diagnosis was seen as an enabler to reach healthier habits because it prompted people to make changes and set goals, “*like before I'm 50 I want to lose weight*” or “*I want to live to 100* years *old*.”

However, some found it “*boring with healthy foods*” or “*food can become a conflict at home*” *or* “*it's hard to maintain the weight loss*” and “*it's difficult to change lifestyle*.” Thus, it is hard to maintain the motivation for behavior change.

#### 4.2.2. I Must Manage It Myself, but I Need Support

People with prediabetes described the choices they make concerning their eating habits, physical activities, stress awareness, sleep, and blood glucose monitoring, as different strategies to self-manage their prediabetes. To accomplish that, they expressed the need for internal support (from family and peers) and external support (from healthcare professionals) describing how this support impacted the sustainability of these changes. This theme was identified from the subthemes: *self-supported self-management* and *healthcare professionals' support or nonsupport.*

##### 4.2.2.1. Self-Supported Self-Management

Persons with sufficient knowledge about prediabetes (both from healthcare professionals and other sources like the internet), support from family and peers, and regular follow-up did not feel the need for further support.

[When asked if they had enough information on prediabetes and support from healthcare] Yes, I think so. I got a lot of information leaflets to read at home. Yes. And now I am followed up once or twice a year at the health center to see how I'm doing. Besides, I got one …. the machine to test [blood glucose monitor]…. (Person 21)

Moreover, some participants expressed having already established healthy habits growing up, such as food and exercise, “*the family has always eaten healthy*,” so they did not need to make any major changes, which contributed to not require any further support than they already had.

Some experienced that self-management could be tricky because there were always some barriers to achieving a healthier lifestyle “*life gets in the way*,” “*easy to lose track if something happens*,” and even financial difficulties. This created a feeling of uncertainty not about the changes they needed to make but about how to make these changes, thus leading to the need for more support from healthcare.

But I need to know how, how to eat healthy and so on… And my daughter is still at home. Now she [buys home]…buns or something [else]tasty and sweet…How do I say no? (Person 12)

##### 4.2.2.2. Healthcare Professionals' Support or Nonsupport

Persons with prediabetes were able to express what kind of support they needed to be able to achieve behavioral changes. This was voiced in the form of information about prediabetes, concrete advice about choices for healthier food and physical activities, reinforcement of any behavioral changes, and follow-up to sustain these changes. When people with prediabetes felt that healthcare professionals provided reassurance and clear information, it was easier to follow the advice and maintain the changes they achieved.

…[Diabetes nurse] gives me reassurance in the information she provided and then I continue…. I have been more consistent [with healthier food and exercise] since I met [the diabetes nurse] …. (Person 9)

The feedback participants received from healthcare professionals was not always positive and encouraging. Sometimes it was focused on what the individual did not attain instead of reinforcing what he/she achieved.

…I'm struggling…so considering what I do… that I exercise a lot, I'm not overweight, I eat healthy food, I kind of don't get a bit of credit for it. … It feels like you can't do anything... like a glass of juice... you can't cross the line, eat something that's not good, and drink something that's not good. They [healthcare professionals] should focus a little more on what you do well. Encourage a little more, don't put down. (Person 3)

The lack of positive feedback and focus from the healthcare on the nonachievement rather than the achievements could explain why some participants used accusive expressions to describe when they treated themselves to “nonhealthy snacks” on rare occasions and referred to that as “*indulging themselves*,” “*sinning*,” and “*cheating*,” which sometimes led to having guilty feelings and other negative effects on self-management.

No, it's as if you decide to take hold of it, so to speak. Then, maybe you eat some things once in a while where you know you are sinning, you feel like “no, I'll do it this time but it must not become a habit”…. Yes, it's a bit of a guilty conscience, you can't get away from it. (Person 1)

They wished for more positive feedback because one is more motivated to continue. Follow-up was therefore important because then the results would be reinforced.

I also see a result every time I come to [the diabetes nurse] that that blood sugar levels did not increase to having type 2 diabetes. But I have managed to keep it at bay. Also, it gives me the strength to continue. (Person 9)

When there was no follow-up, some participants even felt that healthcare professionals did not care about them, and they felt invisible. This could have been reflected in self-management.

They had told me a bit like so, but they left me like that…. So, no one called me, no one. (Person 11)

## 5. Discussion

Persons with prediabetes perceived prediabetes as a condition in between health and disease, along a spectrum from no concern, to taking it very seriously because of the risk of developing Type 2 diabetes and its complications. This in-between state could lead to prediabetes having a low priority when other illnesses or life events take precedence. Thus, one of the main findings of this study showed that it is important to communicate the diagnosis of prediabetes clearly and provide knowledge about preventive measures, as this can trigger behavioral change. Another finding was the support persons with prediabetes needed to carry out optimal self-management and maintain the changes accomplished. This support was described either internally from family and peers or externally from healthcare professionals.

How healthcare professionals communicate prediabetes diagnosis could have a profound impact on persons' responses to having prediabetes and their consequent actions. Previous studies have shown that prediabetes has a low priority in primary healthcare [15–19], guidelines about prediabetes are vague [20], and there is no international consensus on prediabetes diagnosis criteria and treatment [1, 2, 20]. This can create uncertainty about prediabetes diagnosis, treatment options, and follow-up among healthcare professionals. This uncertainty in turn leads to persons with prediabetes being indecisive to take it seriously or not. If healthcare professionals do not convey the risk of prediabetes and the importance of preventive care, it will unlikely be a priority for people with prediabetes as well to take action to prevent Type 2 diabetes.

This further highlights the need for communicating the risk of prediabetes to initiate assertion to act. Other studies [11, 21] have shown the importance of communicating the risk of prediabetes and that prediabetes diagnosis leads to action. The results in this study indicated that when the prediabetes diagnosis was communicated with certainty and with all that it implies to have prediabetes, people with prediabetes took it seriously. They related it to Type 2 diabetes and its complications and could see the opportunity to change behavior to prevent it. Participants could identify what kind of support they needed when reflecting on this.

People with prediabetes expressed that they had to manage prediabetes themselves but needed different support to maintain behavioral change. The internal support from family and peers was expressed as positive support, but for some, it could also be negative. Studies [22, 23] on family support have addressed the importance of this because most of the self-management happens in the family environment. If there is not enough internal support, there will be more need for external support for self-management from healthcare. This was also seen in the study by Skoglund et al. [21] that if resources for the person are low there is a greater need for more support. In a related study [15] where healthcare professionals were interviewed, they discussed the challenges people with prediabetes faced in self-management that were related to the reaction to the diagnosis, knowledge about prediabetes, concrete advice, and more support from family, peers, and healthcare, which coincided with the results from interviews with persons with prediabetes in this study.

However, an additional important support that persons with prediabetes acknowledged in this study was positive feedback from healthcare professionals and the confirmation of their efforts in the changes they accomplished. This positive feedback could minimize the self-stigma that some participants expressed when using accusive expressions. In a cross-sectional study by Kato et al. [24], the results showed the importance of targeting self-stigma and increasing self-esteem and self-efficacy to support behavioral change. Targeting self-stigma leads to increased engagement in self-management. In the metasynthesis study by Skoglund et al. [21], positive health feedback was among the findings that were presented, such as weight loss and improved physical condition, and this positive feedback enhanced self-efficacy. Another key factor was using empowering communication when healthcare professionals facilitated support to persons with prediabetes, which aligns with our findings on positive feedback on results.

People with prediabetes have different perspectives on prediabetes and the support they need to make and maintain behavioral change, as this interview study has shown, which indicates that healthcare professionals need to meet these different needs. This demands a person-centered approach which will be used in the next study in this project when people with prediabetes and healthcare professionals will discuss ideas to support behavioral change targeted to meet the different needs of people with prediabetes.

### 5.1. Limitations and Strengths

One limitation of this study was that the participants came from only four primary care centers in Region Stockholm and therefore cannot shed light on all primary care contexts. Still, the new insights of this study and the results might have relevance for understanding practice beyond our setting; because the participants were from different socioeconomic parts of Region Stockholm, they had a broad range in age and there were almost as many men as women who participated. Another limitation was that we did not collect data regarding participants' socioeconomic status or educational levels which might have had some indirect interpretations to some results. A third limitation was that some interviews were face to face, and others were carried out digitally or by phone, which might have led to a loss of some contextual as well as nonverbal information that could have contributed more to the results.

The strength of this study is that the research team has extensive knowledge of prediabetes and vast experience in qualitative research methodology. In addition, all the interviews were conducted by the same person who had no previous relationship with the participants.

Conducting the interviews was challenging because the people with prediabetes were in an in-between state between disease and health. Thus, they had not reflected on their perception of prediabetes previously. On the other hand, our data have shed light on the challenges of a condition that is not well articulated either by persons with prediabetes or healthcare professionals.

## 6. Conclusions

Perceiving prediabetes as an in-between health and disease state has a serious impact on the decisions that people with prediabetes make concerning self-management and behavioral changes. Thus, it is crucial how healthcare professionals communicate the importance of preventive actions so that persons with prediabetes can become more engaged and motivated in making behavioral changes. People with prediabetes have different perspectives on prediabetes and the support they need; therefore, person-centered support from healthcare professionals for persons with prediabetes is essential for them to be able to carry out optimal self-management and maintain the changes accomplished. The results will be used in an upcoming codesign study where healthcare professionals and persons with prediabetes discuss the components of a person-centered model for a behavioral change intervention in primary healthcare.

## Figures and Tables

**Table 1:** Participant characteristics.

**Characteristics**	**N** = 21
Age in years, median (max–min)	69 (28–85)
Women/men, *n* (%)	11 (52)/10 (48)
Lives alone, *n* (%)	9 (43)
Married/lives with partner, *n* (%)	12 (57)
Working/retired, *n* (%)	7 (33)/14 (67)
Heredity, *n* (%)	12 (57)
*Reason for detection*	
Cardiovascular diseases/hypertension, *n* (%)	10 (48)
Other disease, *n* (%)	7 (33)
Gestational diabetes, *n* (%)	1 (5)
Health checkup, *n* (%)	3 (14)

**Table 2:** Main themes and subthemes.

**Prediabetes: a condition between health and disease**	**I must manage prediabetes myself but need support**
➢ *The in-between perception of prediabetes* ➢ *Prediabetes is my opportunity not to develop Type 2 diabetes*	➢ *Self-supported self-management* ➢ *Healthcare professionals' support or nonsupport*

## Data Availability

The data that support the findings (individual interview transcripts in Swedish) are not openly available because doing so would violate our Swedish Ethical Review Authority. Data are located in controlled access storage at the Karolinska Institutet and the codes, in English, are available from the corresponding author upon reasonable request. The original contributions presented in the study are included in the article, including Supporting Information 1 Interview guide. Other supporting information and any further inquiries can be directed to the corresponding author.
