# Supplementary material for: Should I Take Prediabetes Seriously or Not: A Qualitative Study on People's Perceptions of Prediabetes
Source: J Diabetes Res. 2025 Feb 12;2025:8063481. doi: 10.1155/jdr/8063481 (PMC11839257; doi:10.1155/jdr/8063481)
Supplement: Supporting Information 1 — Additional supporting information can be found online in the Supporting Information section. Interview guide (open questions for people with prediabetes). [file 8063481.f1.docx]

**Supplement 1**

Interview guide:

Open questions for people with prediabetes:

- Can you describe the situation when you got prediabetes? The event itself…

- What did you think when you found out you have prediabetes? Can you describe. Did you know about prediabetes before diagnosis? How was your prediabetes discovered? When did you get it? Did you get any information, if so what information did you get, tell me a little bit. How much of the information could you use, have you used, tell us.

- Can you tell us what you think you need to be able to stop the development of type 2 diabetes?

- What kind of support have you received? What treatment and follow-up have you been offered?

- What additional support would you like to be able to manage prediabetes in everyday life/in your life? Tell. Have you sought help from anywhere other than health care?

- What opportunities do you see for you to make changes in your lifestyle?

- What do you see as obstacles for you to make changes in your lifestyle?
